# N-methyl-D-aspartate receptor antagonist MK-801 prevents apoptosis in rats that have undergone fetal spinal cord transplantation following spinal hemisection

**DOI:** 10.3892/etm.2014.2029

**Published:** 2014-10-17

**Authors:** QIANG ZHANG, YANG SHAO, CHANGSONG ZHAO, JUAN CAI, SHENG SUN

**Affiliations:** 1 Department of Orthopedics, Beijing Ditan Hospital, Capital Medical University, Beijing 100015, P.R. China; 2 Department of Neurology, The Second Affiliated Hospital, Nanjing Medical University, Nanjing, Jiangsu 210011, P.R. China

**Keywords:** spinal cord injury, MK-801, fetal spinal cord, graft, apoptosis

## Abstract

Spinal cord injury is the main cause of paraplegia, but effective therapies for it are lacking. Embryonic spinal cord transplantation is able to repair spinal cord injury, albeit with a large amount of neuronal apoptosis remaining in the spinal cord. MK-801, an N-methyl-D-aspartate (NMDA) receptor antagonist, is able to reduce cell death by decreasing the concentration of excitatory amino acids and preventing extracellular calcium ion influx. In this study, the effect of MK-801 on the apoptosis of spinal cord neurons in rats that have received a fetal spinal cord (FSC) transplant following spinal hemisection was investigated. Wistar rats were divided into three groups: Spinal cord hemisection injury with a combination of FSC transplantation and MK-801 treatment (group A); spinal cord hemisection injury with FSC transplantation (group B); and spinal cord injury with insertion of a Gelfoam pledget (group C). The rats were sacrificed 1, 3, 7 and 14 days after the surgery. Apoptosis in spinal slices from the injured spinal cord was examined by terminal deoxynucleotidyl transferase-mediated dUTP-biotin nick end labeling reaction, and the expression of B-cell lymphoma-2 (Bcl-2) was measured by immunohistochemistry. The positive cells were quantitatively analyzed using a computer image analysis system. The rate of apoptosis and the positive expression of Bcl-2 protein in the spinal cord neurons in the three groups decreased in the following order: C>B>A (P<0.05) and A>B>C (P<0.05), respectively. This indicates that treatment with the NMDA receptor antagonist MK-801 prevents apoptosis in the spinal cord neurons of rats that have undergone FSC transplantation following spinal hemisection.

## Introduction

Spinal cord injury (SCI) is the main cause of paraplegia, but the effective therapies for it are lacking. Experimental studies have shown that SCI consists of primary and secondary damage. The majority of the primary damage of SCI is due to external mechanical stress (1–5). The degree of secondary damage is influenced by a number of injury-promoting factors including edema, ischemia, increased levels of neurotransmitters, increased intracellular Ca^2+^ levels and the presence of free radicals or nitric oxide (NO). In particular, excitatory amino acids (EAAs) are thought to play an important role in the secondary autodestruction of neural tissue following SCI. EAA levels increase following spinal cord trauma in proportion to the severity of injury and this increase exacerbates paralysis in rats, whereas the administration of antagonists of N-methyl-D-aspartate (NMDA) receptors, which are postsynaptic receptors for EAAs, significantly alleviates this paralysis (6–9).

Studies have shown that embryo spinal cord transplantation is able to repair SCI, although a large amount of neuronal apoptosis remains in the spinal cord, and indicate that the NMDA receptor antagonist MK-801 may reduce the cell death by decreasing the concentration of EAAs and preventing extracellular calcium ion influx (10–13). In recent years, the concept of apoptosis in the central nervous system has been introduced to explain the process of ischemia and trauma. Also, it has been found that some neurons and glial cells are apoptotic following SCI (10,14,15). In addition, the overstimulation of glutamate receptors is toxic to neurons and glial cells and is involved in processes culminating in programmed cell death (3,4). Dizocilpine maleate (MK801) is a potent non-competitive NMDA receptor antagonist that blocks the excitotoxic sequelae of ischemia in tissue cultures and animal models of cerebral ischemia, reduces infarct size and improves neurological outcome (5). The efficacies of several NMDA antagonist drugs have been studied in various models of SCI, conducive SCI or in ischemic lesions of the rat spinal cord (11,12,16–19); however, to the best of our knowledge, studies using MK801 to prevent apoptosis in rats that have undergone fetal spinal cord (FSC) transplantation following spinal hemisection have not been reported. Terminal deoxynucleotidyl transferase-mediated dUTP-biotin nick end labeling (TUNEL) reaction and immunohistochemical analysis of B-cell lymphoma-2 (Bcl-2) were used to investigate the effect of the NMDA receptor antagonist MK-801 on apoptosis in rats that have undergone FSC transplantation to treat spinal hemisection.

## Materials and methods

### Experimental animals

Adult Wistar rats (male and female, 180–250 g) were obtained from Vital River Laboratories (Beijing, China) and used in this study. Seventy-two Wistar rats were randomly divided into three groups: Spinal cord hemisection injury with a combination of FSC transplantation and MK-801 treatment (group A); spinal cord hemisection injury with FSC transplantation site (group B); and spinal cord injury with insertion of a Gelfoam pledget (group C). The rats were sacrificed 1, 3, 7 and 14 days subsequent to the surgery. Six animals from each group were sacrificed at each time-point. The animals were transcardially perfused with heparinized saline (0.9%) followed by a solution consisting of 4% paraformaldehyde in 10 mM phosphate-buffered saline (PBS) solution, pH 7.4. The spinal cord was dissected out, and blocks were prepared for cryostat sectioning. Blocks rostral and caudal to the lesion area and blocks containing the lesion/transplant 1 cm respectively were cut in serial transverse 20-μm sections. The apoptosis of spinal slices from the spinal cord was examined by TUNEL reaction and the expression of Bcl-2 was evaluated by immunohistochemistry. The positive cells were quantitatively analyzed using an image analysis system (Tiger Image Analysis system; ICT Research Institute of Chongqing University, Chongqing, China). The animals used in this study were maintained in accordance with the Guide for the Care and Use of Laboratory Animals published by the U.S. National Institutes of Health (20) and the Policy of Animal Care and Use Committee of the Beijing Ditan Hospital of Capital Medical University (Beijing, China).

### Preparation of spinal cord transplants

Timed-pregnant rats were used to provide embryonic spinal cord transplants for insertion into the injured spinal cord of allogeneic rats. Pregnant female rats were anesthetized at 14 days of gestation (E14). The fetuses were removed individually as donor tissue was required and maintained in sterile culture medium. The FSCs were dissected and 1–3 mm^3^ segments of the cord were prepared for transplantation.

### Transplantation methods

Adult rats were anesthetized with 1% pentobarbital sodium (35 mg/kg body weight, intraperitoneal). The backs of the rats were shaved, and the rats were placed on a stereotaxic frame. A laminectomy was performed at the T12-T13 vertebral level. An incision was made with a fine scalpel blade through the meningeal membranes. Lumbar enlargement spinal cord tissue was removed by vacuum aspiration through the meningeal cut, creating a nearly complete hemisection cavity (2×1×1 mm^3^) sparing only left lateral parts. In group C, the rats were hemisected only and Gelfoam of the same size as the lesion was grafted into the lesion cavity. In group B, a solid piece of FSC tissue, ~2×1×1 mm^3^ in length, was drawn up into a sterile glass pipette and gently placed into the cavity subsequent to hemostasis being achieved. The overlying dura was closed with 9-0 Prolene suture and Durafilm was placed over the lesion site. The paraspinal musculature and subcutaneous tissues were closed subsequently with an absorbable suture. In group A, the rats were treated in the same manner as those in group B and were also injected with MK-801 at a dose of 3 mg/kg by tail vein 30 min and 6 h following the surgical procedures.

### Nissl staining

Staining of paraffin sections of 10 μm after 2 times of xylene and graded alcohol dewaxing to water, then add working fluid dye for 10 mins at 37°C. Then add distilled water for about 30 secs after washing and dehydration of 70% alcohol added in 2 mins, then add 0.01% ethanol and eosin solution under the microscope color separation, color separation after the slice with alcohol dehydration, xylene transparent, finally using DPX sealing sections. The staining results for Nissl bodies and nucleolus were purple blue, pink background. The solution consists of 1% azure II aqueous solution, 1% cresyl violet solution, 0.2 M acetate, 0.2 M sodium acetate, absolute ethyl alcohol, double distilled water. Make a total volume of 100 ml in pH 4.0.

### Fluorescence TUNEL

Frozen 20-μm sections were analyzed according to the instructions provided by the manufacturer of the Fluorescein *In situ* Cell Death Detection kit (Boehringer Mannheim Inc., Mannheim, Germany). The positively labeled cells were identified using light microscopy (Olympus, Tokyo, Japan). An apoptosis index (AI) was used to estimate the fraction of apoptotic cells according to the following formula: AI = number of cells with apoptotic nuclei per low-power field (lpf)/total number of cell nuclei per lpf.

### Immunohistochemical analysis of Bcl-2

Antibodies to Bcl-2 (PR-0257; Santa Cruz Biotechnology, Inc., Santa Cruz, CA, USA) was used according to the methods provided with the streptavidin-peroxidase kit (Zhongshan Goldenbridge, Beijing, China). Positively labeled cells were identified using light microscopy. The positive cells were identified in nine slices (three slices from the lesion area, rostral and caudal segments, respectively), randomly. The positive cells (cells/mm^2^) were quantitatively analyzed using a computer image analysis system (Tiger 920G Image Analysis system, ICT Research Institute of Chongqing University, Chongqing, China).

### Statistical analysis

Quantitative image analysis was performed on sections immunostained for Bcl-2 and in the determination of the AI using the image analysis system. The means and standard errors of each intervention group were calculated, and a one-way analysis of variance was performed. Differences between two groups were examined for significance using the Student’s t-test. P<0.05 was considered to indicate a statistically significant result.

## Results

### Nissl staining

**Figure 1:**
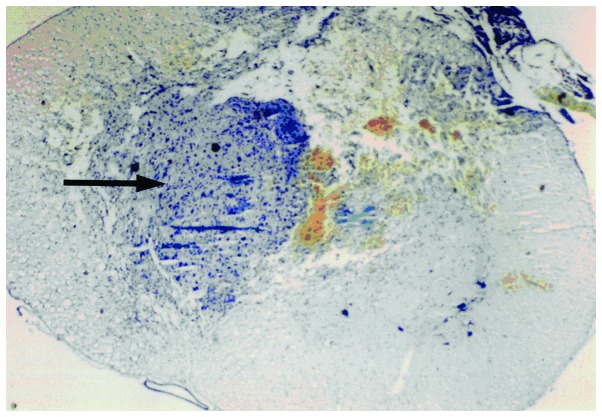
Microphotograph at 14 days after spinal cord injury of a fetal spinal cord tissue transplant. Nissl staining (magnification, ×40). The arrow shows the transplant from the fetal spinal cord tissue.

A microphotograph at 14 days after spinal cord injury of a fetal spinal cord tissue transplant is shown in Fig. 1. The arrow shows the transplant from the fetal spinal cord tissue.

### TUNEL-positive cell counts

**Figure 2:**
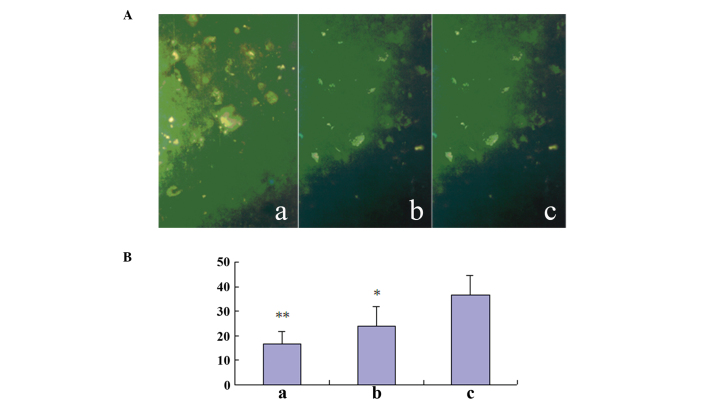
Effect of the N-methyl-D-aspartate receptor antagonist MK-801 on the apoptosis of the ventral horns of gray matter in rats that underwent fetal spinal cord transplantation following spinal hemisection. (A) Representative microphotographs showing TUNEL immunofluorescence staining at 3 days after spinal cord injury (magnification, ×400). (B) Bar graph showing the ratio of the number of positive cells to the total number of cells. Data shown were selected from three independent experiments. ^*^P<0.05 and ^**^P<0.01 vs. group C. a (Group A), spinal cord hemisection injury with fetal spinal cord (FSC) transplantation and MK-801 treatment; b (group B), spinal cord hemisection injury with FSC transplantation; c (group C), spinal cord injury with Gelfoam pledget insertion. TUNEL, terminal deoxynucleotidyl transferase-mediated dUTP-biotin nick end labeling.

TUNEL-positive cell nuclei were observed to be present throughout the gray matter of the spinal cord. Approximately 25% of the spinal cord cells at day 1 were labeled by the TUNEL reaction. At 3 days after SCI, the number of apoptotic cells increased significantly and was at a maximum. These cells were present in the three segments (lesion area, rostral and caudal). From day 7 onward, the number of apoptotic cells gradually decreased. A number of TUNEL-labeled cells were still observed at day 14. Significant differences were identified between the transplantation plus MK-801 treatment (group A), transplantation (group B) and control (group C) groups (Table I; Fig. 2).

### Bcl-2-positive cell counts

**Figure 3:**
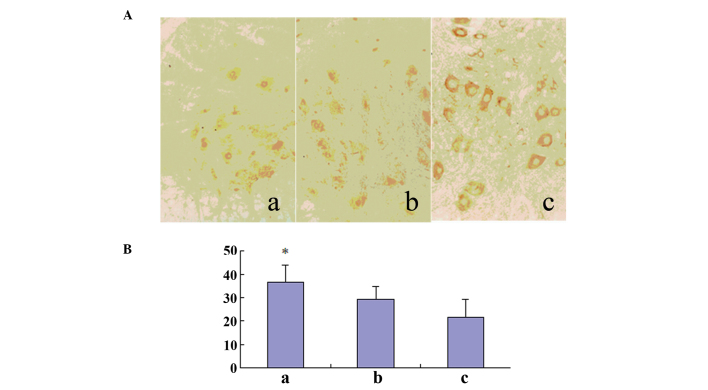
Effect of the N-methyl-D-aspartate receptor antagonist MK-801 on the expression of Bcl-2 in the ventral horns of gray matter in rats that underwent fetal spinal cord transplantation following spinal hemisection. (A) Representative microphotographs showing Bcl-2 immunochemistry with diaminobenzidine staining (magnification, ×200). (B) Bar graph showing the ratio of the number of positive cells to the total number of cells. Data shown were selected from three independent experiments. ^*^P<0.05 and ^**^P<0.01 vs. group C. a (Group A), spinal cord hemisection injury with fetal spinal cord (FSC) transplantation and MK-801 treatment; b (group B), spinal cord hemisection injury with FSC transplantation; c (group C), spinal cord injury with Gelfoam pledget insertion. Bcl-2, B-cell lymphoma-2.

One day after SCI, Bcl-2 immunostained cells (including neurons and glial cells) were detected in the gray matter of the three segments (lesion, upper lesion and lower lesion). The number of positively immunostained cells in groups A and B reached a maximum on day 7. The immunostaining was present in neurons and glia cells. The number of positive cells was maintained at a high level until day 14 and then decreased. The number of Bcl-2-positive cells in Group A was significantly higher than that in the other two groups (Table II; Fig. 3).

## Discussion

Timed-pregnant rats were used to provide embryonic (at 14 days) spinal cord for transplantation into the adult spinal cord lesion site of allogeneic rats. Such transplants can prevent the retrograde cell death of immature axotomized neurons and support the growth of axons into and through the site of injury (21–26). The base of support in animals with transplants has been found to be similar to control values (27). Animals with a hemisection rotate their hindlimbs further laterally than control animals do during locomotion. Spinal cord transplants at the site of adult spinal cord injury (13) result in enhanced sparing or recovery of motor function (28–31). This type of transplant is suggested to induce recovery of function as a consequence of the anatomical plasticity it elicits. The experimental process in which fetuses were removed individually and maintained in sterile culture medium to provide donor tissue, with dissection of the FSCs into 1–3-mm^3^ segments for transplantation, was simple and easy to operate, with good repeatability.

The mechanism by which MK-801 prevents apoptosis was investigated in the present study. Programmed cell death (PCD) is a naturally occurring physiological process that plays a crucial role in the development and maintenance of the brain and spinal cord by eliminating unwanted or unnecessary cells. A number of studies have shown that apoptosis is not only present in the development of the nervous system, but also in trauma and degenerative diseases of the nervous system (32,33,34). Since the 1980s, numerous experimental studies of the treatment of spinal cord injury by FSC have been performed (35,36). It is hypothesized that the transplanted FSC tissue should bridge the spinal lesion and provide chemical and/or mechanical guidance for host neurons to grow across the lesion, bridge the spinal lesion and provide additional cellular elements to repair the damaged circuitry, and provide factors that should rescue neurons that would otherwise die and/or modulate neural circuits to improve function.

Bcl-2 is a unique cytoplasmic protein that is ubiquitous and localized to intracellular sites of oxygen free radical generation, including the mitochondria, endoplasmic reticulum and nuclear membranes. The specific mechanism by which Bcl-2 prevents apoptosis has been investigated. It has been shown *in vitro* and *in vivo* that overexpression of the Bcl-2 oncogene function suppresses lipid peroxidation completely, and thus limits the generation of reactive oxygen species. Thus, it appears that Bcl-2 regulates an antioxidant pathway that limits free radical generation and thereby prevents apoptosis (37–39).

Biochemical and pathological changes in the spinal cord may worsen following injury (40). Necrosis plays a substantial role following injury. In recent years, however, several studies have demonstrated that postinjury spinal cord cell death is due in part to apoptosis, as determined morphologically and biochemically by light and electron microscopic examination, agarose gel electrophoresis and ining (41). Overactivation of EAAs has been shown to induce neuronal degeneration and may contribute to neuronal loss in several disease states. The results of several studies have suggested that exposure to EAAs induces neuronal apoptosis in the brain and in neuronal culture (42,43). NMDA receptor antagonists inhibit neuronal apoptosis in the brain following transient ischemia (44). However, very few studies have been conducted to examine whether EEAs induce neuronal apoptosis in the spinal cord following FSC transplantation. Studies have shown that neurons in the dorsal horn of the spinal cord undergo apoptosis following peripheral nerve insult and that administration of MK-801 reduces the degree of apoptotic cell loss (45). It is suggested that in spinal neurons, apoptosis is induced in a transsynaptic manner by an early signal from injured afferent fibers via activation of spinal NMDA receptors (46). The extrinsic Fas pathway is sufficient to induce complete apoptosis in certain cell types as well as oligodendrocytes, astrocytes and microglia through caspase-8 activation. A further study has demonstrated that treatment with MK-801 significantly reduces Fas ligand positive staining (47). TUNEL-like staining in the perilesional spinal cord tissue was also examined. Almost no apoptotic cells were detected in the spinal cord from sham-operated mice. At 24 h after trauma, tissues from mice with SCI demonstrated a marked appearance of dark brown apoptotic cells and intercellular apoptotic fragments. By contrast, tissues obtained from mice treated with MK-801 demonstrated no apoptotic cells or fragments. This confirmed the well documented neuroprotective effects of MK-801 and lends support to the potential importance of NMDA antagonists as therapeutic agents in the treatment of acute SCI (11,12).

In the present study, the administration of an NMDA receptor antagonist reduced the number of apoptotic cells in white and gray matter in rats that had undergone FSC transplantation following spinal hemisection. This finding suggests that EAAs, which are released from damaged cells, promote delayed cell death due to apoptosis in the spinal cord following injury (34–36). The mechanism by which EAAs induce apoptosis remains unclear. The glutamate-Ca^2+^-NO hypothesis is believed to be the mechanism of EAA-mediated neuronal cell death. According to this hypothesis, the influx of extracellular Ca^2+^ ions is enhanced by EAAs released from damaged cells, resulting in the activation of NO synthase (16–18). Production of NO is then increased in the injured neural tissue. The NO diffuses into the surrounding neurons and glial cells, promotes energy failure, and induces DNA damage in cells. When the injured cells cannot be repaired, delayed cell death may eventually occur due to apoptosis. By contrast, it has been suggested that the excessive Ca^2+^ ion influx activates nuclear endonuclease directly and results in apoptosis (19).

In conclusion, the present study has shown that the NMDA receptor antagonist MK-801 downregulates TUNEL-positive cells and upregulates Bcl-2 positive cells, prevents apoptosis in rats that have undergone FSC transplantation following spinal hemisection and may reduce the toxicity of EAAs. In addition, MK-801 promotes the survival of transplanted FSCs and reduces apoptosis following SCI, consistent with previous studies (39,48).

## Figures and Tables

**Table I:** Apoptosis index following spinal cord injury in rats.

		Days following surgical procedure			
		
Groups	n	1	3	7	14
A	6	12.20±4.52	16.46±5.16^a^	9.58±3.46^b^	7.60±2.50
B	6	15.76±6.84	23.59±7.85^b^	12.54±4.59	10.37±4.52
C	6	28.45±7.81	36.27±8.28	20.96±5.12	9.65±3.56

Group A, spinal cord hemisection injury with fetal spinal cord (FSC) transplantation and MK-801 treatment; group B, spinal cord hemisection injury with FSC transplantation; group C, spinal cord injury with Gelfoam pledget insertion. Data are presented as the mean ± standard error.

a P<0.01 and

b P<0.05 compared with group C.

**Table II:** Bcl-2-positive cells following spinal cord injury in rats (mm^2^).

		Days following surgical procedure			
		
Groups	n	1	3	7	14
A	6	21.57±6.84	34.60±7.53^a^	40.50±8.68^b^	18.36±6.85^a^
B	6	19.85±4.83	29.35±5.38	33.46±6.21^a^	15.35±5.34^b^
C	6	18.56±2.15	21.48±7.83	9.13±3.47	8.87±2.74

Group A, spinal cord hemisection injury with fetal spinal cord (FSC) transplantation and MK-801 treatment; group B, spinal cord hemisection injury with FSC transplantation; group C, spinal cord injury with Gelfoam pledget insertion. Data are presented as the mean ± standard error.

a P<0.05 and

b P<0.01 compared with group C.
